# The effect of massage therapy and/or exercise therapy on subacute or long-lasting neck pain - the Stockholm neck trial (STONE): study protocol for a randomized controlled trial

**DOI:** 10.1186/s13063-015-0926-4

**Published:** 2015-09-16

**Authors:** Eva Skillgate, Anne-Sylvie Bill, Pierre Côté, Peter Viklund, Anna Peterson, Lena W. Holm

**Affiliations:** Musculoskeletal & Sports Injury Epidemiology Center, Institute of Environmental Medicine, Karolinska Institutet, Box 210, SE-17177 Stockholm, Sweden; Naprapathögskolan, Scandinavian College of Naprapathic Manual Medicine, Kräftriket 23A, SE-11419 Stockholm, Sweden; Dalla Lana School of Public Health, Division of Epidemiology, University of Toronto, Toronto, Ontario Canada; Faculty of Health Sciences, University of Ontario, Institute of Technology, UOIT-CMCC Centre for Disability Prevention and Rehabilitation, Oshawa, Ontario Canada; Institute of Health Policy, Management and Evaluation, University of Toronto, Toronto, Canada

**Keywords:** Neck pain, Musculoskeletal manipulations, Manual therapies, Massage, Exercise therapy, Treatment outcome, Complementary therapies/methods, Healthcare costs

## Abstract

**Background:**

Neck pain is a major health problem in populations worldwide and an economic burden in modern societies due to its high prevalence and costs in terms of health care expenditures and lost productivity. Massage and exercise therapy are widely used management options for neck pain. However, there is a lack of scientific evidence regarding their effectiveness for subacute and long-lasting neck pain. This study protocol describes a randomized controlled trial aiming to determine the effect of massage and/or exercise therapy on subacute and long-lasting neck pain over the course of 1 year.

**Methods/Design:**

A randomized controlled trial in which at least 600 study participants with subacute or long-lasting nonspecific neck pain will be recruited and randomly allocated to one of four treatment arms: massage therapy (A), exercise therapy (B), exercise therapy plus massage therapy (C) and advice to stay active (D). The study has an E-health approach, and study participants are being recruited through advertising with a mix of traditional and online marketing channels. Web-based self-report questionnaires measure the main outcomes at 7, 12, 26 and 52 weeks after inclusion. The primary outcomes are a clinically important improvement in pain intensity and pain-related disability at follow-up, measured with a modified version of the Chronic Pain Questionnaire (CPQ). The secondary outcomes are global improvement, health-related quality of life (EQ-5D), sick leave, drug consumption and healthcare utilization. Adverse events are measured by questionnaires at return visits to the clinic, and automated text messages (SMSes) survey neck pain intensity and pain-related disability every week over one year.

**Discussion:**

The results of this study will provide clinicians and stakeholders much needed knowledge to plan medical care for subacute and long-lasting neck pain disorders.

**Trial registration:**

Current Controlled Trials ISRCTN01453590. Date of registration: 3 July 2014.

## Background

Neck pain is a major public health problem because of its high prevalence and costs in terms of healthcare expenditures and lost productivity [1–3]. In the general population, 14 % to 71 % of the adults are affected by neck pain at some point in their lives [4]. The annual prevalence of neck pain varies between 27.1 % and 47.8 % [5]. In the general population in Stockholm, Sweden, 25 % of women and 16 % of men are afflicted by bothersome neck pain each year, peaking in middle age [6]. Between half and three quarters of the persons with current neck pain will report neck pain again 1 to 5 years later [7], and half of all work-related injuries reported in Sweden involve disorders of the muscles and joints [5] The socio-economic impact of neck pain is extensive; it limits workers’ abilities and leads to reduced productivity. It also increases the use of healthcare, leading to a great financial burden [8, 9]. In a report on the global burden of neck pain from 2014, neck pain is considered to be one of the main causes of disability throughout the world, and the authors conclude that neck disability requires greater attention from governments, healthcare providers and researchers [10].

Regarding treatments for neck pain, several nonsurgical management options are available for subacute and long-lasting neck pain disorders. Two commonly used treatment options are massage and exercise therapy. These therapies are widely recommended, prescribed and performed by miscellaneous acknowledged health professionals around the world (such as naprapaths, chiropractors, physiotherapists, osteopaths, massage therapists and personal trainers). Despite their popularity, these therapies have shown incomplete or conflicting evidence of outcomes [11–17] on neck pain.

According to a Cochrane review from 2012, massage therapy for mechanical neck pain was found to provide an immediate or short-term effectiveness or both in pain and tenderness [11]. However, the authors of this systematic review concluded that the evidence for the effect on neck pain is considered inconsistent, and no recommendations for practice can be made for the long-term treatment effect on mechanical neck pain. They stated that future research is needed in order to assess the long-term effects of treatment and treatments provided on more than one occasion. In a systematic review aiming to compare the efficacy, cost-effectiveness, and safety of acupuncture, manipulation, mobilization, and massage in adults with neck or low-back pain, the treatments were considered significantly more efficacious than no treatment, placebo, physical therapy or usual care in reducing pain immediately or for the short-term after treatment. The long-term effects were considered more uncertain [13].

Regarding exercise therapy for mechanical neck disorders, a Cochrane review concludes that neck stretching and strengthening were of benefit in study participants with chronic neck pain for reducing pain and improving function in the short term. However, to confirm these findings, additional studies with larger sample sizes, standardization of treatment dosage and reporting of adverse events are needed [16].

We hypothesize that massage and physical exercise have beneficial effects on subacute and long-lasting neck pain because that is the case with chronic low back pain. There is scientific evidence for the effect of massage on nonspecific low back pain, especially when combined with exercises and education [18, 19]. Physical exercise has been shown to be efficient in reducing the pain and disability in long-lasting low back pain [20, 21] and to reduce work absenteeism [22, 23].

### Study aim

The overall aim of this randomized controlled trial is to determine the effect of massage and/or exercise therapy on subacute and long-lasting neck pain over the course of 1 year.

### Research questions

Specific primary research questions are as follows:

1. Is massage more effective than exercise therapy, a combination of massage and exercise therapy or advice to stay active regarding pain, disability and perceived recovery for persons with nonspecific subacute or long-lasting neck pain?
2. Is exercise therapy or a combination of massage and exercise therapy more effective than advice to stay active regarding pain, disability and recovery for persons with nonspecific subacute or long-lasting neck pain?
3. Is exercise therapy more effective than a combination of massage and exercise therapy regarding pain, disability and recovery for persons with nonspecific subacute or long-lasting neck pain?

Specific secondary research questions are as follows:

1. Is there a difference between massage and/or exercise therapy regarding the risk of adverse events for persons with nonspecific subacute or long-lasting neck pain?
2. What is the course of neck pain over 1 year among persons who gets massage and/or exercise therapy and in persons who gets advice to stay active?
3. What is the cost effectiveness of massage and/or exercise therapy in comparison to advice to stay active?

## Methods/Design

### Study design

The study design is a randomized controlled trial. At least 600 study participants with long-lasting or subacute neck pain will be randomized to one of four treatment arms. To be able to discuss the balance between effectiveness and harm in the treatment arms, adverse events will be monitored with a questionnaire after each visit [24].

A blinded trial is not possible in this case because of the character of the interventions. Attempting to compensate for the lack of blinding, we measure study participant’s previous experiences of the interventions. Furthermore, we ask them about their expectations of recovery before randomization allocation and about their expectations of treatment effect right after finding out the treatment to which they have been allocated.

#### Study population and selection procedure

The trial intends to include persons with subacute or long-lasting neck pain defined as neck pain for at least 30 days impairing daily activities at work and/or leisure time.

The trial has an E-Health approach, and potential study participants are identified by advertising, facilitated through a mix of traditional and online marketing channels. Printed adverts in newspaper are used to draw broad interest to the study, while targeted online adverts are used to reach potential study participants.

### Inclusion criteria

The inclusion criteria are participants who are ongoing neck pain (including whiplash associated disorders (WAD)) and neck pain with headache and/or radiating symptoms in the upper limbs) of subacute (30 to 90 days in duration) or long-lasting (>90 days in duration) duration. The neck pain shall be of such a nature that it is disturbing for daily activities at work and/or leisure time according to the participant in the first contact. The person shall be in the age span 18 to 70 years (Table 1).

**Table 1:** Eligibility screening

Inclusion criteria	Exclusion criteria
Age 18 to 70 years	Not fluent in Swedish language
Current history of subacute neck pain (30 to 90 days duration) or long-lasting neck pain (>90 days) impairing daily activities at work and/or leisure time	Pain intensity <2 as a mean of three pain questions the preceding 4 weeks (Chroniq Pain Questionnaire)
Access to internet	Pain related disability <1 as a mean of three disability questions the preceding 4 weeks (Chronic Pain Questionnaire)
In possession of a smart phone	Having used a personal trainer the preceding 4 weeks
	Pregnancy
	Cancer the preceding 5 years
	Skin disorders in the painful area impeding massage therapy
	Manual therapy, massage therapy or physical therapy for the neck pain in the preceding month
	Sick leave due to surgery in the painful area
	Specific diagnosis (as for example acute prolapsed disc, spondylolisthesis, spinal fracture, spinal stenosis, or arthritis)
	Red Flags (as for example acute trauma, cancer, infection, cauda equine, osteoporosis, or vertebral fractures)
	Fever

### Exclusion criteria

Participants are not included if any of the following criteria are present: 1) not fluent in the Swedish language; 2) mean neck pain intensity <2/10 in the preceding 4 weeks on the three pain questions from a modified version of the Chronic Pain Questionnaire (CPQ); 3) mean pain related disability <1/10 in the preceding 4 weeks on the three disability in the CPQ; 4) pregnancy; 5) cancer in the preceding 5 years; 6) skin condition or fever, which disables the massage; 7) having received treatments for the current complaint by any manual therapist (such as naprapath, chiropractor, osteopath, physiotherapist, or massage therapist); 8) having used a personal trainer during the past month; 9) not having a smart phone and access to the internet; 10) on sick leave due to surgery in the painful area; 11) specific diagnosis such as acute prolapsed disc; 12) spondylolisthesis and spinal stenosis; or 13) “red flags” (older than 55 when the pain debuted for the first time, recent trauma in the area, constant pain or pain getting worse in the night, consumption of steroids now or recently, or drug abuser) [25] (Table 1).

### Recruitment process

When potential study participants contact the study administration, an experienced study assistant informs them about the study and its participation before proceeding to the first step of exclusions and the questions in first baseline questionnaire. The baseline questionnaire is divided into two parts (A and B): the first is completed at the first contact (on the phone) starting with the informed consent for the enrollment in the study, and the second is completed at the first visit to the study center. The baseline questionnaires cover personal information, sociodemographic factors, the most important prognostic factors for neck pain, as well as baseline measurement of the nonretrospective outcomes in the study. A description of the baseline and outcome measurements is presented in Table 2. Participants fulfilling the criteria for participation are sequentially numbered and scheduled for an appointment at the study center located in a clinic at the center of Stockholm.

**Table 2:** Summary of outcomes and assessment schedule

Questionnaire (q)	Amount	Administered by	Week
Baseline			
A	1	Phone/Web	0 (before randomization)
B	1	Paper	0 (before randomization)
Follow-up			
Follow-ups Questionnaire	4	Email	7, 12 ,26 and 52
Follow-up questions	104	Automated text message (SMS)	Two SMSes once a week during one year
Adverse events Qustionnaire	Max 6	Paper	In group A, B and C at each return visit, and the last one by phone

At the first visit to the study center, potential study participants meet one of approximately 15 experienced massage therapists who also are personal trainers, trained to perform the treatments in the trial. Upon arrival, potential study participants receive written information about the study and complete Baseline B. The therapist then performs a physical examination (approximately 15 minutes), using a standardized web-based medical form. The therapist makes exclusion if needed according to Table 1. Finally, the therapist will reveal the study treatment to which the participant has been randomized.

If a therapist excludes a potential study participant due to red flags or undiagnosed specific conditions, the excluded person is encouraged to seek care from a physician as soon as possible.

### Randomization procedure

Included study participants are randomly assigned by block randomization to one of four groups in block sizes of 160 (Table 3). Stratified randomization was not used. Prior to the study start, an assistant not involved in the project prepared 800 opaque, sequentially numbered sealed envelopes with cards marked; 1 (massage), 2 (exercise therapy), 3 (massage and exercise therapy) or 4 (advice) indicating one of the four treatment arms. The number sequence was attained by placing 160 (40 of each numbered fold cards) in an urn, mixing them before placing them, one at the time, into numbered opaque envelopes. Study participants are sequentially numbered by the study assistant before the initial visit, and receive the assigned set of documents, including the envelope, with the corresponding number when they come to the study center. The unmasking is performed by the therapist after the baseline B questionnaire is filled in and after the physical examination and second- step exclusions, so that the study administrator, the therapist and the study participant are blinded to the group assignment until after all study participant baseline data have been collected.

**Table 3:** Interventions

Treatment arm	Description	Time per treatment	Number of sessions	Duration
Massage (A)	Massage will be applied to the neck, thoracic spine area, TMJ^a^ (if indicated) and chest (including the chest muscles)	30 min (in total 45 min session)	up to 6	6 weeks
Exercise therapy (B)	Self-mobilization exercise (gentle controlled movement) of the neck and shoulder joints, including neck retraction, extension, flexion, rotation, lateral bending motions, and scapular retraction) and strengthen exercises	30 min (in total 45 min session)	Up to 6	6 weeks
Massage and Exercise therapy (C)	Combination of A + B	50 min, 25 min of each (in total 60 min session)	Up to 6	6 weeks
	Massage treatment followed by physical exercise.			
Advice to stay active (D)	Support and advice on staying active and on pain coping strategies, according to guidelines and evidence-based reviews	30 min	Up to 3	6 weeks

^a^TMJ: temporomandibular joint

### Sample size

Power analyses based on the primary outcomes have been performed to determine the sample size. A total of 600 participants indicated a power of >80 % to detect a relative risk of 1.2 to 1.3 for a clinically important improvement in pain and disability; a two-step decrease in the mean pain intensity score (CPQ; NRS 0 to 10) and a one-step decrease in mean pain-related disability score (CPQ; NRS 0 to 10) when baseline is compared to the values at follow-up. These calculations are based on the differences in effect between manual therapy and advice to stay active detected in our previous trials, which are 16 to 27 % depending on the length of the follow-up time [26, 27].

### Treatment arms

All treatments are adapted to the study participants’ condition and performed in three different intensities. The intensity level is based on physical examination and the therapist’s clinical experience, as well as after careful communication with the study participant.

#### Massage therapy

Massage therapy can be defined as a technique for manipulating soft tissues using pressure and traction and is applied to the neck, and/or back spine area, chest musculature and jaw-area while the participant lays in supine and prone positions for a session of 30 minutes (total visit time 45 minutes) [28]. The massage treatment is composed of four techniques of soft tissue manipulations. The treatment begins with 1) effleurage, a light stroking technique delivered with moderate pressure, 2) followed by kneading, a firm motion involving compression and subsequent pressure release from the muscle. Then, 3) myofascial trigger points (maximum of six per treatment) are applied with pressure, and eventually, 4) deep muscle/fascia massage technique is used. We use manual trigger point diagnosis, and a trigger point is defined as a palpable small nodule within a taut band of skeletal muscle that reproduces the typical pain, with or without radiation when palpated.

The massage shall be experienced as substantial and beneficial, but not more painful than 5 on a numerical rating scale (NRS) of 0 to 11. Participants randomized to this treatment arm receive up to six treatment sessions over a 6-week period, adapted to the study participant’s condition, with the exact number of visits based on the clinical progress as determined by the therapist and based on their findings and discussion with the study participant.

#### Exercise therapy

Participants randomized to exercise therapy get instruction and support on physical exercises in 30-minute sessions (total visit time 45 minutes) at the study center. The primary focus is on strengthening of the deep neck muscles and shoulder muscles and on strengthening and passive stretching of breast muscles and jaw muscles. The delivery method is one-on-one, and the only exercise equipment used is a rubber band (for the shoulder muscle strengthening. The program is delivered in three intensity levels, individualized and adapted to each participant’s abilities, tolerance, condition and activities of daily living. Participants will be instructed to do three times 10 repetitions of each exercise and 30 seconds of passive stretching when applicable, with a technique of high quality, and to perform the exercise program one to two times per week at home. With the intention of supporting the study participants in performing the exercises at home, the participants are filmed with their own smart phone while doing their specific training program at the study center. Participants randomized to this treatment arm receive up to six treatment sessions over a 6-week period, adapted to the study participant’s condition, with the exact number of visits based on the clinical progress, as determined by the therapist, based on their findings and discussion with the participant.

#### Massage and exercise therapy

Participants randomized to massage and exercise therapy get both the massage and the exercise therapy intervention: massage treatment (25 minutes) followed by exercise therapy (25 minutes of the total visit time 60 minutes) as described above. Participants randomized to this treatment arm receive up to six treatment sessions over a 6-week period, adapted to the study participant’s condition, with the exact number of visits based on the clinical progress as determined by the therapist and based on their findings and discussion with the participant.

#### Advice to stay active

Participants randomized to this treatment arm get advice and support from the therapist. The advice is defined as support and advice on staying active and on pain-coping strategies, according to guidelines and evidence-based reviews [27, 29–31]. The first session is given in direct conjunction to the clinical examination (an additional 15 minutes) by the therapist at the clinic. The aim is to empower the study participant with the understanding of the importance of staying active and living as normal a life as possible, including work and physical activities, and to improve pain coping strategies. Advice on exercises will be general and adapted to the study participant’s condition. A booklet that describes this way of relating to the neck pain and with facts about neck pain and strategies for controlling and preventing pain and for improving quality of life is provided to the study participants [32]. Up to two return visits are offered.

The interventions are summarized in Table 3.

### Baseline

Baseline clinical and demographic data, as well as potential prognostic factors for neck pain, are measured using two baseline questionnaires administered before medical examination, inclusion and randomization. The first baseline questionnaire (A) is completed by telephone interview a maximum of  7 days before the first visit to the study clinic by a research assistant. Study participants fill in the second baseline questionnaire (B) at the study clinic before they meet the therapist at the first visit and before the randomization. The baseline factors are sex, age, education, pain and disability in neck and back [31, 33–35], duration of neck pain, previous episodes of neck pain, health-related quality of life (EQ-5D) [36–38], psychological distress (GHQ-12) [39], smoking, physical exercise, height, weight, sick leave, physical demands at work [40] and job strain [41, 42]. An overview of the Baselines A and B assessment is presented in Table 4.

**Table 4:** Baseline assessments

Baseline	Contains	Instruments
Baseline A (phone call maximum 7 days before first visit)		
General Information [39, 62]	Informed consent for the enrolment in the study	
	Sex	
	Housing and housing environment	
	Education	
	Occupational class [63]	
	Previous experience from massage therapy and/or personal trainer (YES/NO)	
Neck pain	Pain intensity and disability related disability in neck [35]	Chronic Pain Questionnaire
	Duration of neck pain	
	Previous episode of neck pain	
Baseline B (paper questionnaire at first visit)		
Lifestyle [36, 39]	Height and weight	
	Cigarette smoking [64]	
	Leisure time and occupational physical activity [65]	
	Physical demands at work [40, 41]	
General health	Self-rated health [66]	
	Health related quality of life [38]	EQ-5D
	Psychological distress [67]	GHQ-12
	Emotional well-being	
	Anxiety, worry, anguish [68]	
	Expectation of recovery	
	Sleeping problems [42]	
	Persistent fatigue	
Chronic diseases	Diabetes	
	Cardiovascular disease	
	Chronic obstructive pulmonary disease	
	Psoriasis	

**Fig. 1:**
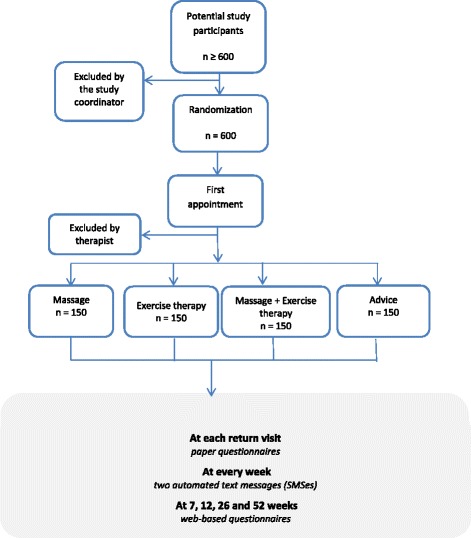
Study procedures

As part of the physical examination study participants undergo a number of clinical neck tests in order to rule out cervical radiculopathies [43], to evaluate the range of motion of the cervical and thoracic spine and to assess the deep cervical muscle function [43–47]. A flow chart over the study procedure is presented in Fig. 1.

### Follow-up

Study participants are followed by web-based questionnaires 7, 12, 26 and 52 weeks after inclusion in the trial and by weekly automated text messages (SMSes) [48–51].

#### Primary outcomes

The primary outcomes are pain and disability, measured by a slightly modified Chronic Pain Questionnaire (CPQ) (4-week recall period instead of 6 months) [31, 33–35] with six items on a numerical 11-point rating scale and one item on the number of disability days of pain and disability. Three items are used to rate the pain (the current pain, the worst pain experienced during the preceding 4 weeks, and an average of the pain during the preceding 4 weeks). A pain score will be constructed from the mean of these three items. Three items are used to rate disability and concern to what degree pain “interfered with your daily activities,” “changed your ability to take part in recreational, social, and family activities” and “changed your ability to work (including housework)” in the past 4 weeks. The disability score will be the mean of these three items. On the basis of these scales, two dichotomized outcomes will be defined based on what is considered to correspond to a clinically significant improvement: a two-step decrease in pain intensity and a one-step decrease in pain-related disability when baseline is compared to the values at follow-up [52].

#### Secondary outcomes

Secondary outcomes will be global perceived improvement [53], health-related quality of life (EQ-5D) [36–38], sick leave, drug consumption, and healthcare utilization. Adverse events within 24 hours post-treatment will be measured at each return visit (duration and severity) with a questionnaire that we have used in a recently published study [24] and was based on the findings reported earlier [27].

#### Automated text message follow-up

The participants are followed up with automated text messages (SMSes) every week to survey the course of neck pain and pain-related disability over one year. The use of automated text messages have been shown to be a valid and cost-effective way to survey low back pain [48, 50, 54]. The questions used are as follows: 1) How much neck pain have you had on average of the past week? Enter a number between 0 (no pain) to 10 (worst pain imaginable). 2) How much has neck pain hindered work/daily activities in the past week? Enter a number between 0 (not at all) to 10 (impossible to perform this).

#### Health economic evaluation

A health economic evaluation will be done aiming to compare costs and outcome between the four treatment alternatives. Diagnosis-related groups (DRG) will be used to define the costs [55], and EQ-5D™ will be used to model the cost effectiveness [56]. As a basis for these analyses, participants will be asked to report healthcare consumption, drug consumption and sick leave days from work in the questionnaires at all follow-ups.

### Statistical analyses

The comparison of the effect between the groups statistical analyses will be performed using an “*intention to treat”* principle aimed at analyzing participants in the group to which they were originally assigned and to keep the dropouts in the assigned group no matter what the reason [57]. To estimate the impact of missing responses, sensitivity analysis for the primary outcomes will be performed using multiple imputation [58]. Changes in mean scores at follow-up compared with baseline, and differences in changes between groups will be calculated by unpaired t-test. To compare the groups regarding the dichotomized outcomes, relative risks (RR) and risk differences (RD), together with corresponding 95 % confidence intervals (CI), will be calculated. Baseline factors that differ between the treatment groups will be considered with regard to their potential confounding effect by means of Mantel Haenszel’s method [59]. If adjustment is needed, the Cox proportional Hazard model will be used.

Generalized estimating equations (GEE) will be performed to analyze the effect on the primary outcome (pain and disability) over the total follow-up time, including the automated text messages (SMSes). The GEE method extends standard regression analysis, taking into account the covariance between repeated measurements of pain and disability [60, 61].

### Timeline

**Fig. 2:**
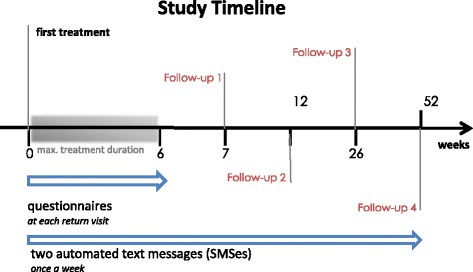
Study timeline

Figure 2 shows the study timeline. Inclusion into this trial started in September 2014. We plan to use 2 years for inclusion of at least 600 study participants and to finish the follow-up data collection during 2017. Data preparation, statistical analyses, manuscript preparation and publication in peer-reviewed scientific journals are planned for 2017 to 2019.

### Ethical considerations

The trial raises ethical issues related to risks of the treatments, the integrity of the study participants, how the collected information will be protected and how research results will be reported. The study collects data from people with symptoms in the neck. The study is based on informed consent, and the information to study subjects clearly states that the study is voluntary and that participants may terminated participation at any time. When collected data are analyzed, personal numbers will be replaced with serial numbers. All collected data will only be used for the purpose of this research, and all in the research group has the ethical principle of confidentiality. The results from the trial will only be presented in tables and figures where no individuals can be identified.

We will process data in accordance with federal guidelines and laws PuL (1998:2004). In addition, we will work actively and continuously to deal with ethical issues that arise during the study. Written informed consent will be obtained from all study subjects.

The treatments are considered safe with very low risk of severe adverse events. We estimate that the benefits of the study will be far greater than any possible risk. The trial has been approved by the Regional Ethics Committee in Stockholm 2014/755-31/3 and is registered at the database Current Controlled Trials (ISRCTN01453590).

## Discussion

Neck pain is one of the most challenging public health issues worldwide. Given its prevalence, the need to assess effective approaches for neck disorders is of prime importance. Although management of non-specific neck pain disorders often includes massage therapy as well as exercise therapy intervention or promotion, little is known about the effectiveness, side effects and cost-effectiveness of such therapies. Scientific evidence supports such therapies for the treatment of low back pain, but their effectiveness for neck pain has not been established. This RCT aims to increase the knowledge on the efficiency, cost and safety of these commonly used treatments for neck pain.

### Strengths

The strengths of this trial includes rigorous randomization procedure, long-term follow-up, the use of standardized outcome measures and a large study population enabling subgroup analyses of the effect of the interventions. The weekly measure of neck pain intensity and neck pain-related disability enables not only a careful follow-up of the effect of the interventions, but also a detailed description of the course of neck pain during a 1-year period, which is an important knowledge for planning of healthcare and secondary prevention strategies. Given the study design, the measurement of the cost-effectiveness of such therapies will be possible. In order to be able to discuss the balance between efficacy and harm, adverse events will be measured (duration and severity) in direct conjunction to the interventions in a standardized way, as described in previous studies of our group on manual therapy [24, 26, 27].

### Limitations

One of the major methodological difficulties inherent to studies evaluating physical interventions is blinding of therapists and study participants. The nature of the interventions makes it impossible to blind the care provider and difficult to blind the study participant.

Unmasking will be performed by the care provider after the physical examination, the second-step exclusions and the data collection with baseline questionnaires, so the study administrator, the therapist and the study participant all will be blind to the group assignment until after all baseline data is collected. We will measure study participant’s previous experience of the interventions, expectation of recovery and expectation of the importance of the allocated therapy for the recovery (just after the unmasking and before the intervention starts). In an attempt to minimize bias from potential differences in expectations of recovery between the four groups will test the potential confounding effects of these factors in the statistical analyses.

## Trial status

The manuscript reports the protocol for an ongoing trial, for which patient recruitment is currently ongoing. The first study participant was included in September 2014 and by May 2015, 340 study participants had been included.

## Abbreviations

CPQ: Chronic Pain Questionnaire
NRS: Numerical rating scale
GEE: Generalized estimating equations
RR: Relative risk
RD: Risk differences
CI: Confidence intervals
